# Navigating the implementation gap for Trastuzumab's journey from health insurance to patient access: a preliminary study in a hospital in China

**DOI:** 10.1186/s41256-024-00384-9

**Published:** 2024-11-26

**Authors:** Xingxia Yang, Yufei Jia, Jianhong Xu, Qin Zhou, Qian Long, Yi Yang, Yunguo Liu, Juanying Zhu, Xiaochen Zhang

**Affiliations:** 1 Department of Breast, Affiliated Women and Children Hospital, Jiaxing University, 2468, East Zhonghua Road, Nanhu District, Jiaxing, 314000 Zhejiang China; 2 Global Health Research Center, Duke Kunshan University, Kunshan, Jiangsu China; 3 Department of General Surgery, Affiliated Kunshan Hospital of Jiangsu University, Kunshan, China; 4 Duke Global Health Institute, Duke University, Durham, NC USA; 5 Division of Social Sciences, Duke Kunshan University, No. 8 Duke Avenue, Kunshan, 215316 Jiangsu China

**Keywords:** Breast cancer, Trastuzumab utilization and accessibility, FISH test, Basic medical insurance coverage

## Abstract

**Background:**

Trastuzumab, a monoclonal antibody for breast cancer, faces global accessibility challenges, primarily due to high costs. This study examines how changes in medical insurance policies and price adjustments influence Trastuzumab utilization in China, focusing on implementation challenges and their impact on drug accessibility and affordability.

**Methods:**

This retrospective study investigated Trastuzumab utilization among HER2-positive breast cancer patients at a tertiary hospital from May 2013 to December 2022 in JX, a prefecture-level city in eastern China. Patients were stratified based on their CerbB2 indicator, because those with a 2 + CerbB2 status require further FISH testing to confirm Trastuzumab eligibility. We analyzed the utilization rates of both FISH test and Trastuzumab using logistic regressions, identifying factors that influence their usage.

**Results:**

766 patients were included. The utilization rate of Trastuzumab among patients with CerbB2 3+ rose from 40.40 to 77.94% in September 2017 following the expansion of insurance coverage, and further increased to 90.41% after a price reduction in 2020. The FISH test was not covered by health insurance, and it was not available in the local hospital until Trastuzumab became available in JX city. For patients with CerbB2 2+, the proportion undergoing the FISH test increased dramatically from 8.89 to 82.08% after the price reduction in 2020. The mere inclusion into basic medical insurance, regardless of insurance types, significantly increased the utilization of Trastuzumab and the FISH test. However, rural patients in JX city are still facing financial burdens with Trastuzumab's out-of-pocket cost accounting for 62.9% of their annual disposable income in 2020.

**Conclusions:**

Our findings indicate that insurance coverage and price reduction significantly increased Trastuzumab utilization. However, failure to improve the accessibility of the FISH test can pose challenges in enhancing the uptake of Trastuzumab among eligible patients. With the expiration of Trastuzumab's patent and the development of affordable biosimilars, there are now greater opportunities to enhance treatment access globally. These insights can inform policy makers of implementation guidance about providing financial support for breast cancer patients in other LMICs.

**Supplementary Information:**

The online version contains supplementary material available at 10.1186/s41256-024-00384-9.

## Background

Breast cancer is the most diagnosed cancer and stands as the leading cause of morbidity in women globally [1, 2]. About 2.3 million women were diagnosed with breast cancer with 685 thousand deaths worldwide in 2020, and the number of new cases was estimated to reach 2.74 million in 2030 if current trends remain unchecked [3, 4]. Among breast cancer patients, those exhibiting overexpression of Human Epidermal Growth Factor Receptor 2 (HER2), accounting for approximately 20–25% of the cases, tend to have a poorer prognosis compared to patients who are HER2-negative [5–7]. Trastuzumab, a monoclonal antibody, signifies a major advancement in the targeted therapy of HER2-positive breast cancer. It is broadly employed in combination with chemotherapy and/or other targeted therapies to improve survival outcomes [8–10].

However, studies have shown that the high cost of Trastuzumab has severely limited its use worldwide, especially in low- and middle-income countries (LMICs). Many healthcare systems struggle to afford this expensive treatment, which leaves many eligible patients without access. One study highlighted the increased risk of recurrence for HER2 + breast cancer patients in parts of Latin America where patients have limited access to anti-HER2 therapies [11]. Additionally, significant discrepancies in Trastuzumab use were observed across the European Union and the US, with some regions in Eastern and Southern Europe facing greater barriers due to economic constraints [12, 13]. These examples highlight the need to overcome cost barriers to ensure more equitable access to this therapy worldwide.

In China, breast cancer is also the most common cancer among females [14, 15]. In 2008, Trastuzumab was registered by the China Food and Drug Administration for the treatment of early-stage HER2-positive breast cancer [16]. In 2008, only 20.17% of HER2-positive patients in Beijing received Trastuzumab treatment [17]. Furthermore, a study spanning 155 hospitals across 29 provinces revealed that merely 29.8% of HER2-positive patients were treated with Trastuzumab between 2013 and 2014 [18]. The high cost of Trastuzumab, potentially resulting in unaffordable out-of-pocket (OOP) expenses for patients and the limited availability were significant factors that restrict its use [14].

To alleviate patients' financial strain from anticancer medications, Trastuzumab injection, marketed as Herceptin, was added to the basic medical insurance reimbursement list in September 2017 following national price negotiations [19–22]. The government-led basic medical insurance is the main body of the medical security system covering over 95% of the population in China [23]. Basic medical insurance includes two systems, the “Urban Employee’s Basic Medical Insurance” (UEBMI) for employees in cities, and “Urban and Rural Resident Basic Medical Insurance” (URRBMI) for urban non-working populations and rural residents [24]. The average reimbursement rate for inpatient costs of medicines on the reimbursement list during 2018 to 2022 was about 80% for UEBMI and 70% for URRBMI [25]. Before reimbursement, the cost of each dose stood at $1129 (with 1 USD equating to 6.73 CNY) after September 2017. Following subsequent negotiations in 2019, the price was reduced to $817, effective nationwide since January 2020 [22]. The percentage of HER2-positive breast cancer patients receiving Trastuzumab treatment at a provincial-level hospital in Central China rose to 58.62% in the year following its inclusion in medical insurance in 2017, up from 32.91% in the preceding year [26]. However, a nationwide survey conducted in 2021 on breast cancer patients revealed that financial toxicity remains a prevalent issue among patients, with the receipt of targeted therapy being a significant risk factor for this financial burden [27]. In addition, there exists a gap in the analysis of the policy implementation process and its consequential effects on the accessibility and uptake of medications such as Trastuzumab.

This study aimed to assess the utilization of Trastuzumab among breast cancer patients, both before and after its inclusion in basic medical insurance and aligned with the price reduction. Additionally, the study sought to explore the role of unique factors, such as the accessibility of the FISH test, in adopting this targeted therapy. We examined and discussed factors that facilitate or hinder the effects of policy implementation. These findings provide valuable insights for other LMICs striving to improve the accessibility of anti-breast cancer drugs and to offer financial risk protection for breast cancer patients.

## Methods

This study focuses on a site in Zhejiang for a case analysis, examining the impact of a step-wedge policy implementation intended to enhance the accessibility and affordability of Trastuzumab.

### Study site

The City of JX, located in Zhejiang Province in the east region of China, is a well-developed city with 5.55 million residents and an annual per capita GDP of $18,097 in 2022 [22, 28]. We selected this hospital in JX for two key reasons. First, JX has implemented several policy changes related to breast cancer treatment, making its data suitable for a pilot study to inform broader policy decisions. As an economically strong city, JX has the financial capacity to promote Trastuzumab usage. Second, the hospital in JX maintains a well-organized database, enabling the collection of accurate and comprehensive data, which allows us to track trends over the study period. The implementation of the policy regarding Trastuzumab's utilization in JX can be tracked through three key milestones:

(i) Integration into medical insurance (October 2017): Before Trastuzumab was covered by the basic medical insurance, its cost per dose in Zhejiang Province was $2890 [22]. After Trastuzumab was added to the medical insurance coverage in October 2017, a volume-based pricing strategy led to a significant price reduction, dropping the cost to $1129 per dose.
(ii) Local availability (March 2019): Following the expansion of insurance coverage, the Trastuzumab treatment in tertiary hospitals achieved reimbursement rates of 80% under UEBMI and 65% under URRBMI in JX city [29]. However, Trastuzumab did not become available in JX city until March 2019; until then, patients had to buy the drug from Hangzhou, the provincial capital of Zhejiang Province, located approximately 55 miles away from JX city. The FISH test was not covered by health insurance, and it was not available in the local hospital until Trastuzumab became available in JX city.
(iii) Price reduction (January 2020): Following its initial inclusion in the insurance, the price of Trastuzumab was further reduced to $817 per dose starting January 2020, making the treatment even more affordable.

### Study design and participants

This study involved a retrospective examination of medical records from the JX city University Affiliated Women and Children Hospital, a tertiary hospital in a city located in eastern China, targeting breast cancer cases treated from May 2013 to December 2022. The hospital has a team of 17 dedicated specialists in breast cancer treatment, supported by a standalone breast specialist ward. It has established a comprehensive breast cancer screening network to enhance early detection rates for both breast cancer and occult breast conditions, ensuring women receive integrated and professional screening, diagnosis, treatment, and follow-up care. The outpatient clinics cater to over 40,000 patients annually, with about 3500 admissions each year. They perform more than 500 breast cancer surgeries in the year 2022. The data from this hospital are representative of the region's healthcare settings, making the findings applicable to similar contexts in other areas within the region.

We included patients who could potentially benefit from Trastuzumab, focusing on those with a potentially positive HER2 status, as indicated by CerbB2 levels of 2+ or 3+ in their pathological reports following breast surgery or core needle biopsy. To ascertain eligibility for Trastuzumab treatment, patients showing a 2+ CerbB2 status should undertake further Fluorescence in Situ Hybridization (FISH) testing to confirm HER2’s expression. In contrast, a 3+ CerbB2 status directly qualified patients for this targeted therapy. Patients diagnosed with ductal carcinoma in situ were excluded from this analysis since, according to the guidelines, they are ineligible for Trastuzumab, even if their CerbB2 status was 3+. The study received ethical approval from the Duke Kunshan University ethics committee (IRB Number: FWA00021580).

### Data collection

Data were extracted from the medical record system from the hospital. We extracted key social and demographic data (age, occupation, education level, pregnancy and childbirth history, and type of medical insurance), diagnosis (pathological type and stage, whether the FISH was administered), and treatment of breast cancer (inpatient and discharge dates, whether trastuzumab was received) for each patient from the database.

### Data analysis

To explore the patterns of Trastuzumab treatment and FISH test utilization among breast cancer patients, we stratified the patients into two groups according to their CerbB2 indicator: those with 3+ and those with 2+ . Chi-square tests were conducted to examine if there were significant differences between patients with CerbB2 2+ and 3+ status in demographic and clinical characteristics. We then examined the percentage of patients with a CerbB2 2+ status who underwent FISH testing, as well as the rates of Trastuzumab treatment among patients with a CerbB2 3+ status and those who tested positive on the FISH test.

Additionally, to assess the influence of health insurance policy shifts and price modifications on the usage of Trastuzumab and the FISH test, we analyzed patient data across different timelines. Patients were grouped based on their discharge dates to coincide with three specific time points: (i) October 2017, marking the conclusion of the drug promotional period following its coverage by medical insurance; (ii) March 2019, signifying the introduction of Trastuzumab in JX city; and (iii) January 2020, denoting the reduction in the price of Trastuzumab to $817 per dose.

Logistic regression models were employed to identify factors influencing Trastuzumab usage among patients either with a CerbB2 rating of 3 + or those with positive FISH test results, as well as the factors affecting the decision to undergo a FISH test among patients rated CerbB2 2 + . The dependent variable was binary, indicating whether patients received Trastuzumab treatment or underwent a FISH test. The independent variables included age, occupation, education level, medical insurance type (UEBMI, URRBMI, and self-paid), pathological stage, number of childbirths, and periods marking the basic medical insurance coverage, treatment availability in JX city, and price reduction of Trastuzumab. Statistical analyses were executed using Stata 16 software.

## Results

### Characteristics of the patients

A total of 766 female breast cancer patients were ultimately included in this study. Table 1 revealed a predominant age group of 45–54 years (39.95%), with an average age of 54.11 years (SD = 10.36, range 21–92 years) at diagnosis. A significant majority (68.02%) had an education of junior high school or below, and the vast majority were married (87.86%). Regarding medical insurance, 46.34% were covered by Urban Employee Basic Medical Insurance (UEBMI), with 41.38% of data missing. Most patients have one child (57.96%) according to their birth history. Discharge records from 2013 to 2022 indicated a peak in 2019 (24.67%). TNM staging of the participants revealed that the largest group was in Stage I (42.56%), followed by Stage II (38.90%). A smaller percentage was in Stage III (13.84%). The demographics between patients with different CerbB2 results did not have significant differences, while most patients with CerbB2 2 + were diagnosed after 2019. Overall, the demographic profile indicated that the patient group consists of individuals who are in their middle to late years, with a basic level of education, married, and identified with early-stage disease at the time of diagnosis. Additional file 1: Table ST1 presents the population distribution of patients with positive FISH test results or CerbB2 3 + across various characteristics. Additional file 1: Table ST2 summarizes the percentage of patients with CerbB2 2 + after Trastuzumab was available in JX city. We found their population distribution is consistent with that of the total study sample.

**Table 1:** Summary of demographic and socioeconomic characteristics of participants

	Total, % (n = 766)	CerbB2 2 + , % (n = 495)	CerbB2 3 + , % (n = 271)	P-value
*Age at diagnosis*				
< 35	2.61	2.83	2.21	0.086
35–44	12.14	11.72	12.92	
45–54	39.95	38.18	43.17	
55–64	30.16	29.49	31.37	
≥ 65	15.14	17.78	10.33	
Mean (range, SD)	54.11 (21–92, 10.36)	54.78 (26–92, 10.90)	52.89 (21–77, 9.18)	
*Educational Level*				
Junior high school or below	68.02	67.07	69.74	0.294
High school or technical school	6.53	7.47	4.8	
Bachelor's or associate degree	6.27	6.67	5.54	
Missing	18.79	19.92	18.79	
*Marital Status*				
Married	87.86	86.06	91.14	0.777
Divorced	0.52	0.4	0.74	
Widowed	0.91	1.01	0.74	
Single	0.65	0.81	0.37	
Missing	10.06	11.72	7.01	
*Medical insurance*^1*^				
UEBMI	46.34	38.99	59.78	0.066
URRBMI	7.57	8.28	6.27	
Self-pay	4.7	4.04	5.9	
Missing	41.39	48.69	28.05	
*Number of Childbirths*				
0	1.83	2.02	1.48	0.181
1	56.14	52.53	62.73	
2	28.2	28.48	27.68	
> = 3	3.52	16.97	1.48	
Missing	10.31	0	6.63	
*Time of discharge from hospital*				
2013	4.96	6.87	1.48	< 0.001
2014	7.05	5.25	10.33	
2015	7.44	7.07	8.12	
2016	5.35	4.24	7.38	
2017	6.53	3.64	11.81	
2018	11.88	7.68	19.56	
2019	24.67	30.3	14.39	
2020	17.36	18.18	15.87	
2021	9.79	11.52	6.64	
2022	4.96	5.25	4.43	
*TNM Stage*^2^				
0	1.04	0.81	1.48	0.684
I	42.69	44.24	39.85	
II	38.90	38.18	40.22	
III	13.84	14.75	12.18	
IV	0.26	0.2	0.37	
Missing	3.27	1.82	5.9	

1. *UEBMI* Urban Employee Basic Medical Insurance; *URRBMI* Urban and Rural Residents Basic Medical Insurance

2. The TNM system is utilized to characterize cancer's extent and spread in a patient's body. It encompasses T for tumor size and local spread, N for lymph node involvement, and M for metastasis. Developed and maintained by the American Joint Committee on Cancer (AJCC) and the International Union Against Cancer (UICC), this system is widely employed across various cancer types

### The utilization of the Trastuzumab Treatment and FISH Test

**Fig. 1:**
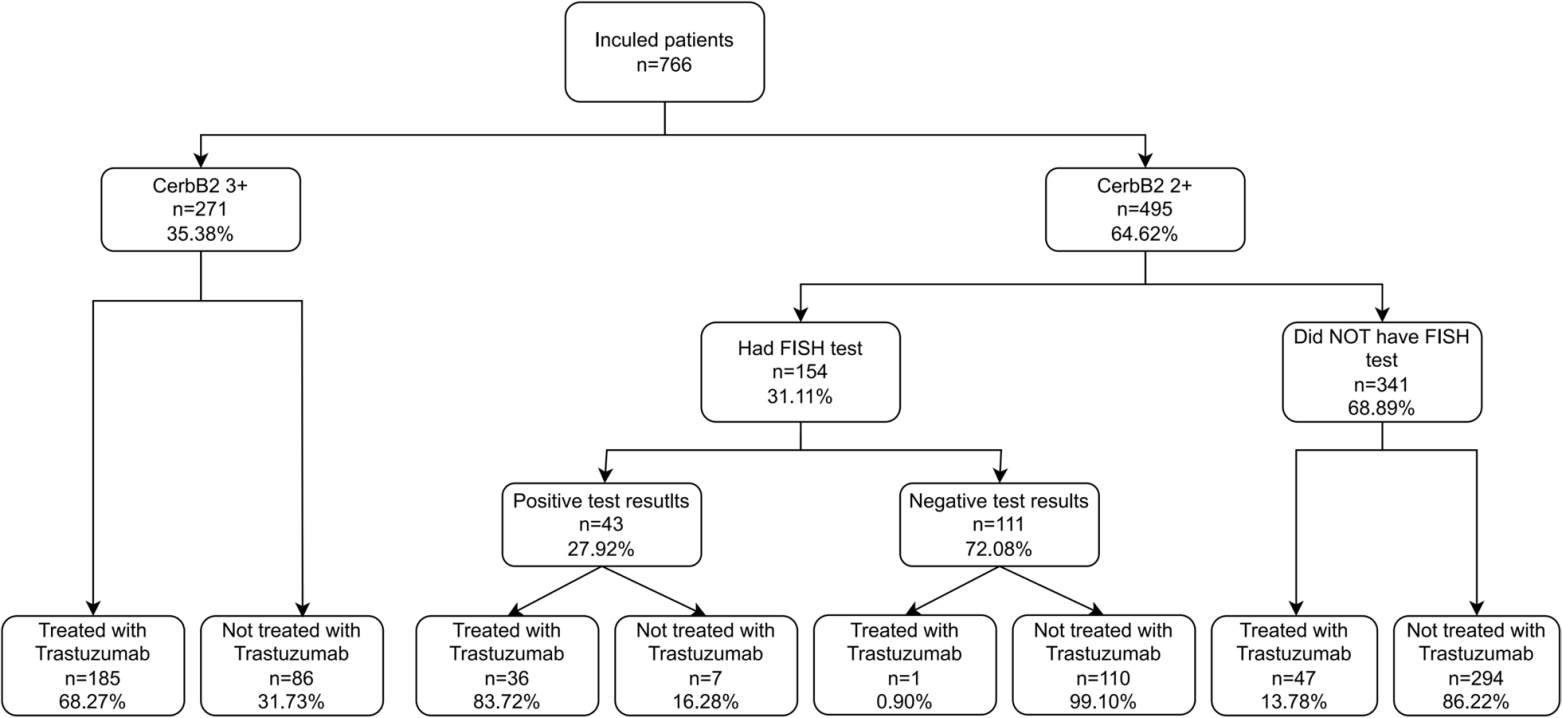
Utilization of Trastuzumab Treatment and FISH Testing among Participants

Figure 1 shows how Trastuzumab was used among all the patients in our study. Initially, 35.38% were found to be HER2-positive, making them eligible for Trastuzumab; of these, 68.27% started the treatment, while 31.73% did not. The remaining 64.62% of patients needed additional testing, but only 31.11% actually took the FISH test. Among those tested, 27.92% had positive results, and 83.71% of these patients opted for Trastuzumab, a higher uptake than among initially identified patients. In total, 314 patients were eligible for Trastuzumab based on CerbB2 or FISH results, and 221 of them received the treatment. However, 68.89% of those requiring further testing did not get the FISH test, and most did not receive Trastuzumab. Notably, 13.78% of these patients still received Trastuzumab, suggesting potential testing outside of our documented system.

Table 2 illustrates the changes over time. Initially, only 40.40% of the identified eligible patients received the treatment before it was covered by insurance. With insurance coverage, the figure rose to 77.94% even before it was largely available at the local hospitals. Following the availability of the medicine in JX city, the treatment rate increased to 83.87% among 31 identified eligible patients. After the price reduction, the rate further climbed to 90.41% among 73 eligible patients, the highest observed in the study.

**Table 2:** Temporal Trends in FISH Test and Trastuzumab Utilization

	Total % (n = 766)	Before covered by basic medical insurance	Covered by insurance but not available in JX city	Available in JX city	Price reduced to $817 per dose
		Before Oct 2017 (n = 227)	Oct 2017-Feb 2019(n = 127)	Mar 2019-Dec 2019 (n = 166)	After Jan 2020 (n = 246)
Identified Trastuzumab-eligible patients (CerbB2 3 +)	n = 271	n = 99	n = 68	n = 31	n = 73
Treated with Trastuzumab	68.27	40.40	77.94	83.87	90.41
Not treated with Trastuzumab	31.73	59.60	22.06	16.13	9.59
Patients needed further confirmation through FISH tests (CerbB2 2 +)	n = 495	n = 128	n = 59	n = 135	n = 173
Patients had FISH tests	n = 154 (31.11%)	n = 0 (0.00%)	n = 0 (0.00%)	n = 12 (8.89%)	n = 142 (82.08%)
Patients with positive results	n = 43	n = 0	n = 0	n = 2	n = 40
Treated with Trastuzumab	83.72	–	–	0	87.50
Not treated with Trastuzumab	16.28	–	–	100	12.50
Patients did NOT have FISH tests	n = 341 (68.89%)	n = 128 (100.00%)	n = 59 (100.00%)	n = 123 (91.11%)	n = 31 (17.92%)
Treated with Trastuzumab	13.78	11.72	18.64	14.63	9.68
Not treated with Trastuzumab	86.22	88.28	81.36	85.37	90.32

The study didn’t collect data on FISH test usage before Trastuzumab was made locally available, as the service was not provided by the local hospital at that time. Patients who tend to use Trastuzumab need to visit the test laboratory to undergo the test independently, rather than through the hospital. Additionally, physicians tended not to inform patients about the test, considering its high cost and the local unavailability of Trastuzumab. Even after Trastuzumab became available in JX city, only 12 out of 135 patients were recorded as having taken the FISH test. Even if accounting for those who received the treatment without recording the test results, which amounts to 14.63% of 123 patients, it is estimated that only about 22.22% of patients possibly had the test before the price drop to 817 USD per dose. However, after the price was reduced, the rate of test participation notably increased to 82.08%.

### The out-of-pocket costs after insurance coverage

Table 3 presents the estimated out-of-pocket (OOP) costs of Trastuzumab for a typical female patient in the years 2018 and 2020. 2018 marks the first year following its inclusion in basic medical insurance, with a cost of $1129 per dose, and 2020 marks the first year when the price reduced to $817 per dose. As per guideline [30], a typical female breast cancer patient in our study, with an average weight of 58 kg, would require 13 doses of Trastuzumab. URRBMI has a lower reimbursement rate than UEBMI. The OOP cost for Trastuzumab decreased from $2935.4 in 2018 to $2124.2 for patients covered by UEBMI, and from $5137.0 to $3717.4 for those covered by URRBMI.

**Table 3:** The Estimation of the OOP Costs for Trastuzumab Treatment in 2016, 2018, and 2020

Time	2018					
Region	Urban		Rural (URRBMI)	Urban		Rural (URRBMI)
	UEBMI	URRBMI		UEBMI	URRBMI	
Price per dose (USD)	1129			817		
Number of doses	13			13		
Reimbursement rate	80%	65%	65%	80%	65%	65%
OOP cost on Trastuzumab (USD)	2935.4	5137.0	5137.0	2124.2	3717.4	3717.4
Annual disposable income per capita (USD)	8534.5		5093.5	9528.1		5914.0
Ratio of OOP cost/annual disposable income per capita (%)	34.4%	60.2%	100.9%	22.3%	39.0%	62.9%

We use the annual per capita disposable income to estimate the financial burden associated with Trastuzumab [31, 32]. Patients covered by UEBMI experienced the lowest ratio of OOP cost to annual disposable income per capita. However, for rural patients, although Trastuzumab was covered by medical insurance, the OOP cost for the drug surpassed the average annual disposable income per capita in 2018. Even after the price reduction, the OOP cost for Trastuzumab still accounted for 62.9% of the average disposable income for rural patients.

### Determinants of Trastuzumab usage

Table 4 shows the characteristics associated with the utilization of Trastuzumab among HER2-positive breast cancer patients. The findings showed that patients over 65 years old, with an odds ratio (OR) of 0.09 (95% CI 0.01–0.52), inversely correlates with the use of Trastuzumab, suggesting they may be less likely to use this treatment. Occupation, level of education, and type of medical insurance did not significantly impact the likelihood of Trastuzumab use. In the regression analysis, basic medical insurance coverage emerged as the only significant predictor of Trastuzumab use, significantly increasing the likelihood of patients opting for this treatment after receiving insurance support (OR 8.65, 95% CI 4.13–18.12, *P* < 0.001). To further reduce concerns about each policy’s impacts, we conducted another round of analysis by doing before and after analysis for both two events—the insurance coverage expansion & the price reduction. Specifically, we conducted regressions on patients treated after health insurance coverage but before the price reduction (from 2013 to 2020) to assess the impact of Trastuzumab’s availability. We also analyzed patients treated after local availability (from 2019 to 2022) to evaluate the effect of price reductions. However, the results presented in Additional file 1: Tables ST1 and ST2, still indicated that neither policy change had a significant impact on the Trastuzumab utilization.

**Table 4:** Characteristics associated with the utilization of Trastuzumab among HER2-positive patients

	Odds ratio (OR)	(95%CI)	P value
Age			
< 35	1.00		
35–44	0.62	(0.12, 3.07)	0.555
45–54	0.59	(0.13, 2.61)	0.484
55–64	0.33	(0.07, 1.56)	0.162
≥ 65	0.09	(0.01, 0.52)	0.008
TNM Stage	1.60	(1.10, 2.32)	0.015
Occupation			
Employed	1.00		
Freelancer or unemployed	0.54	(0.18, 1.64)	0.276
Farmer	4.86	(0.55, 42.86)	0.155
Retired	3.85	(1.53, 9.65)	0.004
Other	1.50	(0.73, 3.08)	0.267
Education level			
Junior high school or below	1.00		
High school or technical school	0.45	(0.16, 1.30)	0.142
Bachelor's degree or associate degree	0.51	(0.17, 1.56)	0.247
Insurance Type			
UEBMI	1.00		
URRBMI	0.57	(0.25, 1.30)	0.181
Self-pay	1.08	(0.41, 2.86)	0.872
Number of childbirths	0.79	(0.49, 1.26)	0.318
**Covered by basic medical insurance**	8.65	(4.13, 18.12)	**< 0.001**

Bold indicates significant at alpha = 0.001 level

Table 5 indicates that the price reduction of Trastuzumab to $817 per dose was the sole factor significantly influencing the utilization of the FISH test (OR 111.78, 95% CI 45.66–273.65, *P* < 0.001) among patients with CerbB2 2 + results. Other variables such as age, TNM stage, occupation, education level, and number of childbirths did not show a significant association with the likelihood of undergoing FISH test.

**Table 5:** Characteristics associated with the utilization of FISH test among patients requiring future confirmation

	Odds ratio (OR)	(95%CI)	P value
Age			
< 35	1.00		
35–44	0.53	(0.04, 6.61)	0.619
45–54	0.98	(0.09, 10.45)	0.986
55–64	0.69	(0.06, 7.84)	0.766
≥ 65	0.60	(0.05, 8.1)	0.704
TNM Stage	1.12	(0.66, 1.91)	0.665
Occupation			
Employed	1.00		
Freelancer or unemployed	2.25	(0.43, 11.75)	0.338
Farmer	3.12	(0.03, 288.4)	0.622
Retired	2.43	(0.52, 11.32)	0.259
Other	0.69	(0.25, 1.93)	0.480
Education level			
Junior high school or below	1.00		
High school or technical school	2.48	(0.66, 9.3)	0.179
Bachelor's degree or associate degree	1.54	(0.35, 6.72)	0.563
Number of childbirths	1.01	(0.57, 1.79)	0.983
**Price lowered to $817 per dose**	111.78	(45.66, 273.65)	** < 0.001**

Bold indicates significant at alpha = 0.001 level

## Discussion

This study emphasizes the significant influence of health insurance policy changes on the utilization of Trastuzumab among breast cancer patients, highlighting the challenges in policy implementation. A significant increase in the use of Trastuzumab was noted after its inclusion in the basic medical insurance reimbursement list. The utilization rate soared to 90% following the reduction in price to $817 per dose for patients with 3 + CerbB2 status. The local availability of Trastuzumab and the reduction in Trastuzumab's price enhanced the accessibility of the FISH test, which plays a critical role in identifying the potential beneficiaries from the targeted therapy. Consequently, over 80% of the patients with CerbB2 2 + status underwent the tests after the year 2020.

### Affordability as a key determinant of Trastuzumab utilization

This study observed that before the inclusion in basic medical insurance, the utilization rate of Trastuzumab among HER2-positive patients in JX city (40.4%) exceeded the national average (29.8%) and that reported by a hospital in central China (32.9%) [18, 26]. However, even in an economically advanced region of China, the financial burden of Trastuzumab on patients was still substantial before the implementation of basic medical insurance coverage. For example, in Jiangsu, another economically advanced province in China, even high-income patients struggle to afford anticancer medications without the support of basic medical insurance coverage on the medicines [33]. Furthermore, the high cost may influence physician behavior, making those at the study hospital hesitant to strongly recommend the therapy.

A significant increase in Trastuzumab utilization was observed following the introduction of basic medical insurance coverage and the price reduction, which is consistent with findings from studies conducted in other Chinese provinces [16, 26, 34]. Our study found that the level of medical insurance coverage is the only factor influencing Trastuzumab utilization in JX city. This is similar to the findings by Shang, indicating that HER2-positive patients covered by both UEBMI and URRBMI didn’t show significant differences in Trastuzumab usage. Instead, the crucial determinant was the availability of basic medical insurance coverage in 2017 [34]. Furthermore, our study revealed a significant rise in utilization rates, reaching approximately 90% in 2022 after a price reduction in 2020. The reduction in financial burden post-reimbursement and lower prices could make anticancer medicines more affordable for patients [33]. Additionally, given the life-threatening nature of cancer, patients may be more likely to bear higher costs for anticancer drugs compared to treatments for other chronic illnesses [33, 35].

However, disparities in financial burden persist between urban and rural regions, as indicated by previous research [27, 36, 37]. While our study focused solely on estimating the OOP cost of Trastuzumab for breast cancer patients, we found that due to higher reimbursement rates and disposable annual income, patients under UEBMI experienced lower financial burdens than those under URRBMI. However, for rural patients, the out-of-pocket cost of completing Trastuzumab therapy at $817 per dose could exceed 60% of the average annual disposable income per person, regardless of other medical expenditures or non-medical costs. Additionally, studies have shown that despite increased utilization rates, over 70% of HER2-positive cancer patients did not complete the treatment after Trastuzumab's inclusion in medical insurance. This could be attributed to a lack of awareness or financial constraints [26]. It could be inferred that while more rural patients may opt to use Trastuzumab, they may still face financial challenges and may not complete the recommended therapy.

### The influence of local Trastuzumab accessibility on treatment choices

The local availability of Trastuzumab also influenced patients' access to the treatment in JX city. For patients with CerbB2 3 + results, there was an increase in utilization rates once Trastuzumab became available in JX city, which emphasized the importance of improving drug distribution networks and ensuring timely access to medications post-policy changes. However, t the influence of local availability was less pronounced than that of insurance coverage, possibly due to the geographic proximity between JX city and Hangzhou, which allows patients and their families to easily obtain the medication from neighboring cities. Previous studies suggest that basic medical insurance coverage and price negotiations have improved the nationwide availability of anticancer medicines in China, yet regional disparities still persist [38, 39]. In eastern China, where our study was conducted, the availability is relatively higher than in the central and western regions [38]. Further studies are warranted to examine the effect of anticancer medication availability throughout the country following the expansion of basic insurance coverage.

### The role of FISH test accessibility in the adoption of targeted therapies

Our analysis highlights the significance of FISH test accessibility in influencing Trastuzumab treatment uptake among eligible candidates. Earlier studies have largely centered on confirmed HER2-positive patients, neglecting those who require additional diagnostic evaluations [16–18, 26]. However, a significant portion (around 65%) of the patients included in our study needed further testing to determine their eligibility for Trastuzumab treatment. These diagnostic tests are deemed necessary for treatment planning, but their accessibility in developing countries are sometimes limited due to budget constraints. If not covered by insurance plans, these tests would require low-income patients to pay out of their own pockets or turn to less expensive alternatives. Data from 2020 to 2022 revealed that nearly 30% of patients who underwent FISH test were found eligible for Trastuzumab, suggesting that without this diagnostic test, a substantial number of breast cancer patients might overlook the opportunity for this targeted therapy.

The FISH test was made available at the study hospital when Trastuzumab was first introduced to the city of JX city. However, since the FISH test is not covered by insurance, patients face OOP expenses of about $500 for the test. Our regression analysis highlights the substantial influence of pricing on the adoption of diagnostic tests, given that test fees are not covered by any form of medical insurance in China. This implies that the already low utilization of Trastuzumab may be exacerbated by patients' limited access to essential diagnostic procedures. The affordability of ancillary services, such as eligibility tests, may also greatly influence patients' healthcare decisions.

### Implications for global health practices

Targeted therapies are dominating anticancer drug spending these days [40]. However, the long duration of trastuzumab use in clinical practice, coupled with the high prices of Trastuzumab, has caused an ongoing debate about the cost-effectiveness of such expensive medicines. For example, some study found that using targeted therapies as a cost-effective strategy in the United States requires at least a 50% reduction in the drug acquisition cost [41]. In Australia, trastuzumab was deemed not cost-effective for treating metastatic breast cancer and therefore was not approved for inclusion on the Pharmaceutical Benefits Schedule, the country's list of government-subsidized medications [42]. In many LMICs, the economic burden of Trastuzumab lead to widely denied access to such effective but expensive treatment [43] For instance, in Brazil, only 5.6% of patients with HER2 + tumors received trastuzumab through the public healthcare system, compared to 56% in the private sector in 2006 [44]. Many barriers to access of Trastuzumab still exist worldwide, especially in LMICs, and the equal access to expensive anticancer medicines, such as targeted therapy, is far from universal.

In China, the inclusion of Trastuzumab in the national reimbursement drug list allowed for a reduction in its price through bulk purchasing mechanisms, significantly increasing its utilization among HER2 + breast cancer patients and lowering their out-of-pocket costs. Globally, the selection of medicines for national essential medicines lists is also a crucial first step toward ensuring access to breast cancer treatments in LMICs. However, less than 10% of 75 LMICs have included Trastuzumab in their lists, primarily due to the financial burden on their health systems [45]. Drug costs have been reported as a barrier to prescribing adjuvant Trastuzumab in LMICs, according to both international physician and patient-reported surveys [46, 47]. Although some countries may not have the same population size as China to leverage bulk purchasing advantages, the availability of biosimilars may create opportunities for health systems to introduce breast cancer-targeted therapies at a lower cost. The patent for Trastuzumab expired in 2020, and the World Health Organization has prequalified biosimilars that are up to 65% cheaper than the originator drug, thus expanding global access to these life-saving treatments [48]. A study in India reported that the use of Trastuzumab biosimilars increased due to their affordability and improved accessibility [49].

As this study revealed, the high cost of the FISH test may also prevent some potential beneficiaries from being identified. Additionally, the limited capacity for conducting molecular diagnostic tests hinders the use of targeted therapies in many LMICs [50]. Our findings highlight the need to improve diagnostic capabilities and expand insurance coverage to enhance access to breast cancer-targeted treatments.

Our study still has some limitations. Firstly, this study is based on data from JX city, which may limit the generalizability of the findings to other regions in China or other countries. However, the challenges identified in this economically advanced area highlight issues that could be even more pronounced in less developed regions, warranting further attention. Secondly, our study is limited by missing data. For example, some patients may have taken their own FISH test through some other channels instead of the hospital studied, making their test results inaccessible and creating gaps in our dataset. Additionally, the absence of insurance type information for certain patients limits our ability to examine the potential heterogeneity across different insurance plans, such as UEBMI and URRBMI. Future research aiming to understand the varying impacts of insurance plans could benefit from more comprehensive data on insurance types. Thirdly, we are unable to control for natural time trends due to the structure of our data, but we can see the notable changes in the percentage of breast cancer using Trastuzumab. Future studies could benefit from controlled experiments to uncover the causal effects of policy changes. Additionally, future research can conduct qualitative studies to understand the real experiences of doctors and patients in prescribing and using the medication. This could provide deeper insights into the barriers and facilitators affecting Trastuzumab utilization.

## Conclusions

Our study provides updated evidence on the effects of medical insurance policy changes on Trastuzumab utilization, particularly by tracing the price and availability changes through a case study in China. Our results show that the mere inclusion of Trastuzumab in insurance coverage, significantly increases patients' utilization, despite the fact that out-of-pocket payments remain burdensome for many. Secondly, our research draws attention to potential beneficiaries by highlighting the critical role of FISH test accessibility in determining Trastuzumab utilization. We recommend that tests essential for determining eligibility of cancer treatments, such as the FISH test for Trastuzumab therapy, should be added to insurance reimbursement lists to enhance patient access. Lastly, our study calls for more attention and policies aimed at reducing the regional disparities between urban and rural areas, as well as those among different regions within the country. These findings are particularly relevant for LMICs where the healthcare systems are usually underfunded. The insurance may only cover standard treatment such as Trastuzumab but exclude coverage for related expensive diagnostic tests like FISH due to cost considerations, which poses a significant challenge in improving patient access.

## Supplementary Information

**Additional file 1.**

## Data Availability

The original data supporting the study's findings are not publicly available due to privacy and institutional policies.

## Abbreviations

HER2: Human Epidermal Growth Factor Receptor 2
LMICs: Low- and middle-income countries
OOP: Out-of-pocket
UEBMI: Urban Employee’s Basic Medical Insurance
URRBMI: Urban and Rural Resident Basic Medical Insurance
UHC: Universal health coverage
GDP: Gross Domestic Product
FISH: Fluorescence in Situ Hybridization
