# Supplementary material for: Navigating the implementation gap for Trastuzumab's journey from health insurance to patient access: a preliminary study in a hospital in China
Source: Glob Health Res Policy. 2024 Nov 26;9:50. doi: 10.1186/s41256-024-00384-9 (PMC11590308; doi:10.1186/s41256-024-00384-9)
Supplement: Supplementary file 1 — Additional file 1. [file 41256_2024_384_MOESM1_ESM.docx]

**Supplementary Tables**

ST 1. Percentage for patients with positive FISH test results or CerbB2 3+

|  | **Total, %**  **(n=326)** |
| --- | --- |
| **Age at Diagnosis** |  |
| <35 | 3.04 |
| 35-44 | 13.26 |
| 45-54 | 43.09 |
| 55-64 | 29.83 |
| ≥65 | 10.77 |
| Mean (range, SD) | 52.78  (21-77, 9.36) |
| **Educational Level ^*^** |  |
| Junior high school or below | 68.23 |
| High school or technical school | 5.52 |
| Bachelor's or associate degree | 6.08 |
| Missing | 20.17 |
| **Marital Status^*^** |  |
| Married | 89.23 |
| Divorced | 0.83 |
| Widowed | 1.1 |
| Single | 0.83 |
| Missing | 8.01 |
| **Medical Insurance^1*^** |  |
| UEBMI | 56.35 |
| URRBMI | 8.29 |
| Self-pay | 6.63 |
| Missing | 28.73 |
| **Number of Childbirths^*^** |  |
| 0 | 1.93 |
| 1 | 62.98 |
| 2 | 25.14 |
| >=3 | 1.66 |
| Missing | 8.29 |
| **TNM Stage^2*^** |  |
| 0 | 1.1 |
| I | 38.67 |
| II | 40.33 |
| III | 14.36 |
| IV | 0.28 |
| Missing | 5.25 |
| **Trastuzumab covered by basic medical Insurance** |  |
| Yes | 31.49 |
| No | 68.51 |
| **Available in JX city** |  |
| Yes | 53.31 |
| No | 46.69 |
| **Price lowered to $817 per dose** |  |
| Yes | 67.4 |
| No | 32.6 |

ST 2. Percentage of patients with CerbB2 2+ after Trastuzumab was available in JX city

|  | **Total, %**  **(n=308)** |
| --- | --- |
| **Age at Diagnosis** |  |
| <35 | 3.57 |
| 35-44 | 11.04 |
| 45-54 | 34.42 |
| 55-64 | 31.82 |
| ≥65 | 19.16 |
| Mean (range, SD) | 55.28  (26-92, 11.21) |
| **Educational Level ^*^** |  |
| Junior high school or below | 64.29 |
| High school or technical school | 7.47 |
| Bachelor's or associate degree | 9.09 |
| Missing | 19.16 |
| **Marital Status^*^** |  |
| Married | 78.57 |
| Divorced | 0.65 |
| Widowed | 0.97 |
| Single | 1.3 |
| Missing | 18.51 |
| **Medical Insurance^1*^** |  |
| UEBMI | 21.1 |
| URRBMI | 13.31 |
| Self-pay | 2.92 |
| Missing | 62.66 |
| **Number of Childbirths^*^** |  |
| 0 | 2.6 |
| 1 | 49.68 |
| 2 | 25.00 |
| >=3 | 3.89 |
| Missing | 18.83 |
| **TNM Stage^2*^** |  |
| 0 | 0.97 |
| I | 46.43 |
| II | 35.06 |
| III | 16.56 |
| IV | 0.00 |
| Missing | 0.97 |
| **Price lowered to $817 per dose** |  |
| Yes | 43.83 |
| No | 56.17 |

ST 3. Characteristics associated with the utilization of Trastuzumab among HER2-positive patients in JX city after its health insurance coverage but before the price reduction (between 2019 and 2022)

|  | **Odds ratio (OR)** | **(95%CI)** | **P value** |
| --- | --- | --- | --- |
| **Age** | 0.52 | (0.29, 0.94) | 0.029 |
| **TNM Stage** | 2.28 | (1.06, 4.91) | 0.035 |
| **Education level** |  |  |  |
| Junior high school or below | 1.00 |  |  |
| High school or technical school | 2.05 | (0.23, 18.29) | 0.520 |
| Bachelor's degree or associate degree | 1.00 | (empty) |  |
| **Number of childbirths** | 1.26 | (0.53, 2.96) | 0.601 |
| **Prive lowered to $817 per dose** | 1.82 | (0.64, 5.20) | 0.265 |

ST 4. Characteristics associated with the utilization of Trastuzumab among HER2-positive patients in JX city after its introduction (between 2013 and 2020)

|  | **Odds ratio (OR)** | **(95%CI)** | **P value** |
| --- | --- | --- | --- |
| **Age** |  |  |  |
| <35 | 1.00 |  |  |
| 35-44 | 0.23 | (0.02, 2.87) | 0.256 |
| 45-54 | 0.63 | (0.07, 5.40) | 0.672 |
| 55-64 | 0.35 | (0.04, 3.43) | 0.369 |
| ≥65 | 0.07 | (0.01, 0.91) | 0.042 |
| **TNM Stage** | 1.46 | (0.80, 2.68) | 0.219 |
| **Occupation** |  |  |  |
| Employed | 1.00 |  |  |
| Freelancer or unemployed | 0.30 | (0.07, 1.18) | 0.084 |
| Farmer | 2.02 | (0.08, 54.02) | 0.676 |
| Retired | 2.31 | (0.74, 7.24) | 0.150 |
| Other* | 0.68 | (0.10, 4.79) | 0.701 |
| **Education level** |  |  |  |
| Junior high school or below | 1.00 |  |  |
| High school or technical school | 1.00 | (empty) |  |
| Bachelor's degree or associate degree | 0.28 | (0.07, 1.15) | 0.077 |
| **Insurance Type** |  |  |  |
| UEBMI | 1.00 |  |  |
| URRBMI | 0.59 | (0.23, 1.52) | 0.278 |
| Self-pay | 0.14 | (0.01, 1.91) | 0.138 |
| **Number of childbirths** | 1.11 | (0.54, 2.28) | 0.781 |
| **Prive lowered to $817 per dose** | 1.18 | (0.20, 6.94) | 0.858 |
